# Cure of Disseminated Human Lymphoma with [^177^Lu]Lu-Ofatumumab in a Preclinical Model

**DOI:** 10.2967/jnumed.122.264816

**Published:** 2023-04

**Authors:** Kyuhwan Shim, Mark S. Longtine, Diane S. Abou, Mark J. Hoegger, Richard S. Laforest, Daniel L.J. Thorek, Richard L. Wahl

**Affiliations:** 1 Mallinckrodt Institute of Radiology, Washington University School of Medicine, St. Louis, Missouri;; 2 Department of Biomedical Engineering, Washington University, St. Louis, Missouri; and; 3 Department of Radiation Oncology, Washington University, St. Louis, Missouri

**Keywords:** CD20, lymphoma, targeted β-particle therapy, radioimmunotherapy, lutetium

## Abstract

Although immunotherapies that target CD20 on most non-Hodgkin lymphoma (NHL) cells have improved patient outcomes, current therapies are inadequate because many cases are, or become, refractory or undergo relapse. Here, we labelled the third-generation human anti-CD20 antibody ofatumumab with ^177^Lu, determined the in vitro characteristics of [^177^Lu]Lu-ofatumumab, estimated human dosimetry, and assayed tumor targeting and therapeutic efficacy in a murine model of disseminated NHL. **Methods:** CHX-A″-diethylenetriaminepentaacetic acid-[^177^Lu]Lu-ofatumumab was prepared. We evaluated radiochemical yield, purity, in vitro immunoreactivity, stability, (*n* = 7), affinity, and killing of CD20-expressing Raji cells (*n* = 3). Human dosimetry was estimated from biodistribution studies as percentage injected activity per gram using C57BL/6N mice. Tissue and organ biodistribution was determined in R2G2 immunodeficient mice with subcutaneous Raji-cell tumors. Therapy studies used R2G2 mice with disseminated human Raji-luc tumor cells (*n* = 10 mice/group). Four days after cell injection, the mice were left untreated or were treated with ofatumumab, 8.51 MBq of [^177^Lu]Lu-IgG, or 0.74 or 8.51 MBq of [^177^Lu]Lu-ofatumumab. Survival, weight, and bioluminescence were tracked. **Results:** Radiochemical yield was 93% ± 2%, radiochemical purity was 99% ± 1%, and specific activity was 401 ± 17 MBq/mg. Immunoreactivity was substantially preserved, and more than 75% of ^177^Lu remained chelated after 7 d in serum. [^177^Lu]Lu-ofatumumab specifically killed Raji-luc cells in vitro (*P* < 0.05). Dosimetry estimated that an effective dose for human administration is 0.36 mSv/MBq and that marrow may be the dose-limiting organ. Biodistribution in subcutaneous tumors 1, 3, and 7 d after [^177^Lu]Lu-ofatumumab injection was 11, 15, and 14 percentage injected activity per gram, respectively. In the therapy study, median survival of untreated mice was 19 d, not statistically different from mice treated with 8.51 MBq of [^177^Lu]Lu-IgG (25 d). Unlabeled ofatumumab increased survival to 46 d, similar to 0.74 MBq of [^177^Lu]Lu-ofatumumab (59 d), with both being superior to no treatment (*P* < 0.0003). Weight loss and increased tumor burden preceded death or killing of the animal for cause. In contrast, treatment with 8.51 MBq of [^177^Lu]Lu-ofatumumab dramatically increased median survival (>221 d), permitted weight gain, eliminated detectable tumors, and was curative in 9 of 10 mice. **Conclusion:** [^177^Lu]Lu-ofatumumab shows favorable in vitro characteristics, localizes to tumor, and demonstrates curative therapeutic efficacy in a disseminated lymphoma model, showing potential for clinical translation to treat NHL.

## Visual Abstract

**Figure:**
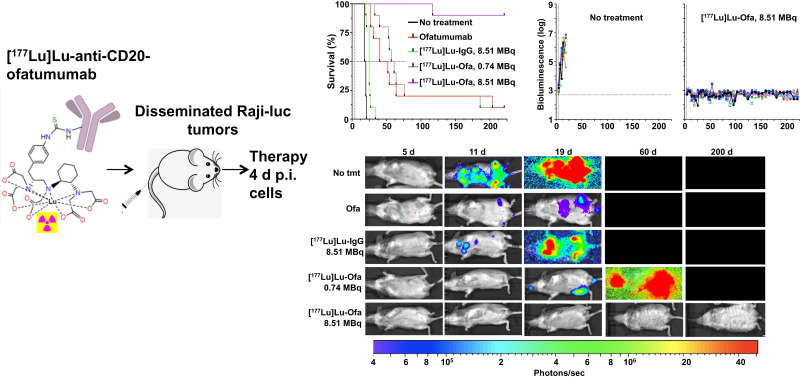

Non-Hodgkin lymphoma (NHL) is a common hematologic malignancy, with over 80,000 new cases and 20,000 deaths estimated for the United States in 2022 (1). The standard of care for many cases of NHL involves chemotherapy and immunotherapy targeting the CD20 protein, which is highly expressed on most NHL cells, with murine/human chimeric rituximab used most commonly. Although this chemotherapy-with-immunotherapy combination is usually initially effective, many cases are refractory or undergo relapse, indicating the need for improved therapies.

Radioimmunotherapy joined clinical practice 2 decades ago with Food and Drug Administration approval of 2 anti-CD20 radioimmunotherapies for lymphoma: Zevalin ([^90^Y]Y-ibritumomab tiuxetan; Acrotech Biopharma, Inc.) and Bexxar (tositumomab and ^131^I-tositumomab; GlaxoSmithKline), which use murine-derived antibodies radiolabeled with β-particle–emitting radioisotopes. Because of potential immune reactions, these antibodies were approved for only a single therapeutic dose. ^90^Y (half-life [t_½_], 2.7 d) emits high-energy β-particles (average, 934 keV), whereas ^131^I emits lower-energy β-particles (average, 187 keV), with average ranges in tissue of 3,800 μm and 360 μm, respectively (2), enabling killing over many cell diameters. Thus, in addition to working against individual tumor cells, β-particles may work against larger tumors, tumor-cell aggregates with imperfect antibody access, and heterogeneous tumors, although with potential off-target damage. Despite long-term safety and clinical effectiveness, Bexxar has been discontinued in the United States and Zevalin is applied infrequently (3), in part because of economic and logistic concerns that were present when they were introduced and because of competing nonradioactive therapies (4).

Some concerns that limited the use of Bexxar and Zevalin have been overcome with greater integration of radiopharmaceutical therapy into medicine (5), as exemplified by the Food and Drug Administration approval of ^177^Lu-labeled agents for prostate cancer treatment (Pluvicto; Advanced Accelerator Applications (6)) and neuroendocrine tumors (Lutathera; Advanced Accelerator Applications (7)). ^177^Lu (t_½_, 6.6 d) emits β-particles of 149 keV on average, with an average tissue range of 220 μm. Emission of low-abundance γ-particles by ^177^Lu permits imaging by SPECT.

Recently, ofatumumab, a third-generation anti-CD20 fully human antibody, was developed. Ofatumumab is a type I antibody that is internalized after CD20 binding (8). We showed by biodistribution and PET imaging studies that [^89^Zr]Zr-DFO-ofatumumab targets CD20-positive subcutaneous xenograft tumors as well as [^89^Zr]Zr-DFO-rituximab (9).

Here, we describe the synthesis and evaluation of [^177^Lu]Lu-ofatumumab. We present in vitro characteristics, dosimetry estimation, and subcutaneous tumor targeting. We also show that [^177^Lu]Lu-ofatumumab therapy results in long-term survival and elimination of tumor cells in a murine model of disseminated human lymphoma.

## MATERIALS AND METHODS

### Reagents and Cell Culture

Ofatumumab (IgG1 κ; Novartis) was purchased from the Washington University clinical pharmacy, and human IgG1 κ was purchased from BioXcel. Raji cells and Raji-luc cells stably expressing luciferase (10) were cultured as previously described (9). SCN-CHX-A″-DTPA ({[(R)-2-amino-3-(4 isothiocyanatophenyl)propyl]-trans(S,S)-cyclohexane-1,2-diamine-pentaacetic acid}) was from Macrocyclics, size-exclusion chromatography columns from Fisher Scientific, and d-luciferin from GoldBio. Sigma provided human serum, sodium acetate, diethylenetriamine pentaacetate, tetramethylammonium acetate, and l-sodium ascorbate. ^177^Lu from the University of Missouri was dissolved in 0.2 M HCl. Silica gel thin-layer chromatography paper was from Agilent, and the 3-(4,5-dimethylthiazol-2-yl)-5-(3-carboxymethoxyphenyl)-2-(4-sulfophenyl)-2H-tetrazolium (MTS) salt assay was from Promega.

### Antibody Conjugation, Radiolabeling, Thin-Layer Chromatography, Mass Spectrometry, and Fast-Performance Liquid Chromatography

Antibody was incubated with SCN-CHX-A″-DTPA in 0.1 M sodium carbonate, pH 9.0, at a chelator-to-antibody molar ratio of 8:1 for 1 h at 37°C and purified by size-exclusion chromatography into 0.5 M NH_4_OAc, pH 7.0. A 477-MBq quantity of ^177^Lu was added to 400 μg of CHX-A″-DTPA-antibody with 20 mM NH_4_OAc, pH 7.0. After 2 h at 37°C, DTPA was added to 5 mM final concentration, followed by size-exclusion chromatography purification into saline and the addition of a 10 mg/mL concentration of l-sodium ascorbate. Thin-layer chromatography and fast-performance liquid chromatography were done as previously described (9). Radiochemical yield was assayed with a CRC55-tW dose calibrator. Chelate number was determined using a Fisher Scientific Exactive Plus EMR mass spectrometer operating at a mass (*m*)-to-charge (*z*) range from 800 to 12,000 and a resolving power of 8,750 or 17,500 at 300* m/z*. Data were analyzed using Protein Metric Intact software.

### Serum Stability, Immunoreactivity, In Vitro Stability, Affinity, and Cell Killing Assays

To assay stability, 14.8 MBq of [^177^Lu]Lu-ofatumumab or ^177^Lu were added to 10% human serum in 20 mM NaOAc 150 mM NaCl pH 7.0 with 10 mg/mL L-SA and incubated at 37°C. Another aliquot of [^177^Lu]-ofatumumab was incubated at 4°C in buffer without serum and with 10 mg/mL L-SA. Aliquots were analyzed by thin-layer chromatography at 0, 1, 5, and 7 d. Immunoreactivity was assayed as previously described (9). To assay affinity, 2.5 × 10^6^ Raji cells without or with 10 μg of ofatumumab were incubated with [^177^Lu]Lu-ofatumumab, washed after 4 h at 23°C, and γ-counted. To assay cell killing, 2 × 10^6^ Raji-luc cells in 1 mL of RPMI medium with 10% heat-treated fetal bovine serum were exposed to no treatment, ofatumumab, [^177^Lu]Lu-IgG, or [^177^Lu]Lu-ofatumumab, with cognate unlabeled antibody added to 20 μg total. After 14 h at 37°C, the cells were washed and 20% were resuspended in fresh medium for an additional 168 h followed by MTS assay.

### Biodistribution of [^177^Lu]Lu-Ofatumumab in Mice with Subcutaneous Raji Tumors

The Washington University in St. Louis Animal Care and Use Committee approved the animal studies. Biodistribution with tumor-bearing mice used female 6- to 8-wk-old immunodeficient Rag2-IL2rg (R2G2, B6;129-*Rag2^tm1Fwa^II2rg^tm1Rsky^*/DwlHsd) mice (Envigo) injected subcutaneously with 5 × 10^6^ Raji-luc cells. Mice with palpable tumors were injected intravenously with 10–20 μg of [^177^Lu]Lu-ofatumumab and killed 1, 3, or 7 d later. Distribution was calculated as decay-corrected percentage injected activity per gram (%IA/g) using a Beckman 8000 γ-counter and a 1- to 500-keV window.

### Dosimetry Estimation

Naïve 5- to 6-wk-old C57Bl6/N mice injected intravenously with 370 kBq (10 μg) of [^177^Lu]Lu-ofatumumab were killed 4 h, 1 d, 2 d, 5 d, or 11 d later, and tissue and organs were γ-counted. Bone was counted after marrow separation. Urine and feces were collected at 4 h, 1 d, and 2 d. Organ residence times were calculated by analytic integration of single or multiexponential fits of the time–activity curve and scaled to human organ weight by relative organ mass scaling (11), which was not applied to the gastrointestinal tract organs. To estimate human radiation dose, residence times were entered into OLINDA, version 2.2, using the MIRD adult-female model and organ weights from International Commission on Radiological Protection publication 106 (12). The calculated radiation dose includes contributions from β- and γ-rays from ^177^Lu within the organ, neighboring organs, and remainder of the body.

### Therapeutic Studies, Mouse Weight, and Bioluminescent Imaging

R2G2 mice (10 per group) injected intravenously with 1 × 10^6^ Raji-luc cells and either left untreated or injected 4 d later with ofatumumab, [^177^Lu]Lu-IgG, or [^177^Lu]Lu-ofatumumab. When used, 20 μg of antibody were injected per mouse. Bioluminescent images were acquired as previously described (13). Mice were killed if they experienced hind-limb paralysis, lost more than 20% of their body weight, or had other signs of morbidity.

### Statistics

Statistical analyses used Prism software (version 9.0; GraphPad).

## RESULTS

### Synthesis of [^177^Lu]Lu-Ofatumumab and Radiochemical Yield, Purity, and Immunoreactivity

SCN-CHX-A″-DTPA was conjugated to ofatumumab and purified. Mass spectrometry indicated an average of 3.2 chelators per antibody. After ^177^Lu radiolabeling, [^177^Lu]Lu-ofatumumab was purified (*n* = 7). Radiochemical purity was more than 99% ± 1%, radiochemical yield was 93% ± 2%, and specific activity was 401 ± 17 MBq/mg. Immunoreactivity was 49% ± 3% and 2% ± 1% after blocking with unlabeled ofatumumab.

### Serum Stability, In Vitro Cell Killing, and Affinity of [^177^Lu]Lu-Ofatumumab

After 7 d, over 90% of ^177^Lu remained chelated in buffer at 4°C, and over 75% remained chelated in human serum at 37°C (Supplemental Fig. 1A; supplemental materials are available at http://jnm.snmjournals.org). Targeting and killing of CD20-expressing cells were assayed (Supplemental Fig. 1B) by adding either no antibody or [^177^Lu]Lu-ofatumumab or [^177^Lu]Lu-IgG (0.74–11.10 MBq/mL) to Raji-luc cells; incubating for 14 h; changing the medium; and, 168 h later, determining cell viability. Compared with no antibody, [^177^Lu]Lu-IgG showed no cell killing at any dose. [^177^Lu]Lu-ofatumumab at 3.7 MBq/mL or higher showed dose-dependent killing compared with no antibody and [^177^Lu]Lu-IgG (*P* < 0.05, *n* = 3). [^177^Lu]Lu-ofatumumab showed a 4.3 nM dissociation constant for CD20 (Supplemental Fig. 1C), consistent with that noted previously (as described for 2F2 by Teeling et al. (14)).

### Biodistribution of [^177^Lu]Lu-Ofatumumab in C57Bl6/N Mice and Estimation of Human Dosimetry

[^177^Lu]Lu-ofatumumab biodistribution was determined in C57Bl6/N mice 4 h, 1 d, 2 d, 7 d, and 11 d after injection (Supplemental Table 1) as %IA/g. Blood %IA/g was 38% at 4 h and 19% after 11 d. Bone distribution was less than 4%, indicating stable chelation because free ^177^Lu is a bone-seeking radionuclide (15). Liver was 9 %IA at 4 h and 5 %IA/g at 11 d, and marrow was 14 %IA at 4 h and 9 %IA/g at 11 d. Spleen was 8–9 %IA/g. Approximately 13% of the injected activity was excreted.

To estimate human dosimetry, integrated time–activity curves for [^177^Lu]Lu-ofatumumab were calculated (Supplemental Table 2). The longest (59.7 h) was in the blood, with extended time–activity curves seen in the blood-rich heart cavity, lung, and liver. Because of its large mass, muscle had the second longest time–activity curve, at 39 h. The adult human female model (Table 1) showed estimated dosimetry of 0.2–0.5 mSv/MBq in most organs, with the largest dose being to the heart wall (1.02 mSv/MBq) and lesser doses found for liver, spleen, and kidney (0.36, 0.48, and 0.43 mSv/MBq, respectively). Estimated doses to the osteogenic cells (bone surfaces) and red marrow were 0.82 and 0.54 mSv/MBq, respectively. The estimated effective dose was 0.36 mSv/MBq.

**TABLE 1.** Human Radiation Dose Estimates for [^177^Lu]Lu-Ofatumumab Extrapolated to Adult Female Model

Organ	mSv/MBq	rad/mCi
Adrenals	0.39	1.44
Brain	0.05	0.19
Breasts	0.25	0.91
Esophagus	0.26	0.96
Eyes	0.25	0.91
Gallbladder wall	0.28	1.02
Left colon	0.36	1.34
Small intestine	0.41	1.50
Stomach wall	0.29	1.09
Right colon	0.29	1.08
Rectum	0.26	0.97
Heart wall	1.02	3.77
Kidneys	0.43	1.60
Liver	0.36	1.32
Lungs	0.53	1.96
Ovaries	0.39	1.44
Pancreas	0.21	0.77
Salivary glands	0.25	0.92
Red marrow	0.54	2.01
Osteogenic cells	0.82	3.02
Spleen	0.48	1.76
Thymus	0.26	0.96
Thyroid	0.80	2.97
Urinary bladder wall	0.34	1.27
Uterus	0.54	2.00
Total body	0.31	1.14
Effective dose (mGy/MBq; rem/mCi)	0.36	1.34

### Biodistribution of [^177^Lu]Lu-Ofatumumab in Mice with Subcutaneous Raji-Cell Tumors

**FIGURE 1.**
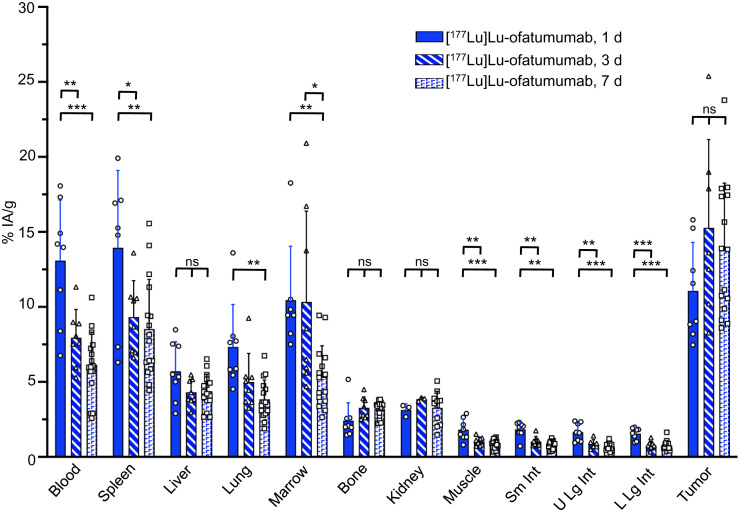
[^177^Lu]Lu-ofatumumab biodistribution in R2G2 mice with subcutaneous Raji tumors. Biodistribution was assayed 1, 3, or 7 d after radiopharmaceutical injection (3–16 mice per time point), with data presented as mean ± SD. One-way ANOVA compares distribution in organ or tissue at each time point. *P < 0.05. **P < 0.001. ***P < 0.0001. Sm Int = small intestine; U Lg Int = upper large intestine; L Lg Int = lower large intestine.

Biodistribution was investigated in R2G2 mice with subcutaneous Raji-cell tumors (Fig. 1). These mice are proficient in double-strand DNA-break repair and are less likely to show artifactual radiation toxicity than are repair-deficient *Prkdc^SCID^* mice (16). [^177^Lu]Lu-ofatumumab was injected at a low activity (370–444 kBq) to limit therapeutic effect, and biodistribution was determined 1, 3, and 7 d later (3–16 mice per time point). Blood decreased from about 13 to 6 %IA/g, with a similar splenic distribution. Liver levels were about 5%, and marrow was 10 %IA at 1 d and 5 %IA/g at 7 d. Bone distribution was 2–3 %IA/g. Tumor targeting was 11, 15, and 14 %IA/g at 1, 3, and 7 d, respectively.

### Murine Therapy Study

To evaluate [^177^Lu]Lu-ofatumumab therapeutic efficacy, R2G2 mice were injected intravenously with Raji-luc cells, and tumor cells were quantified by bioluminescent imaging (13). After injection, these cells disseminate to many organs (10*,*^13^*,*^17^*,*^18^), with hind-limb paralysis being a typical cause for killing of the animal due to growth in and around the spine.

**FIGURE 2.**
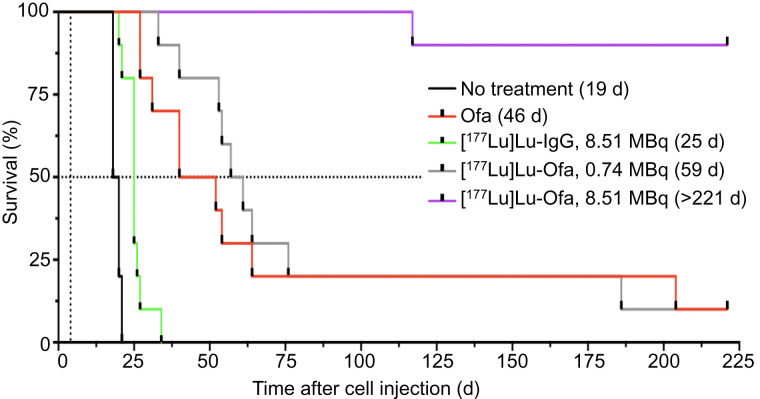
Survival analysis of mice with disseminated Raji-luc cells with therapy initiated 4 d after cell injection. Kaplan–Meier graph shows median survival, in days. Ofa = ofatumumab.

**FIGURE 3.**
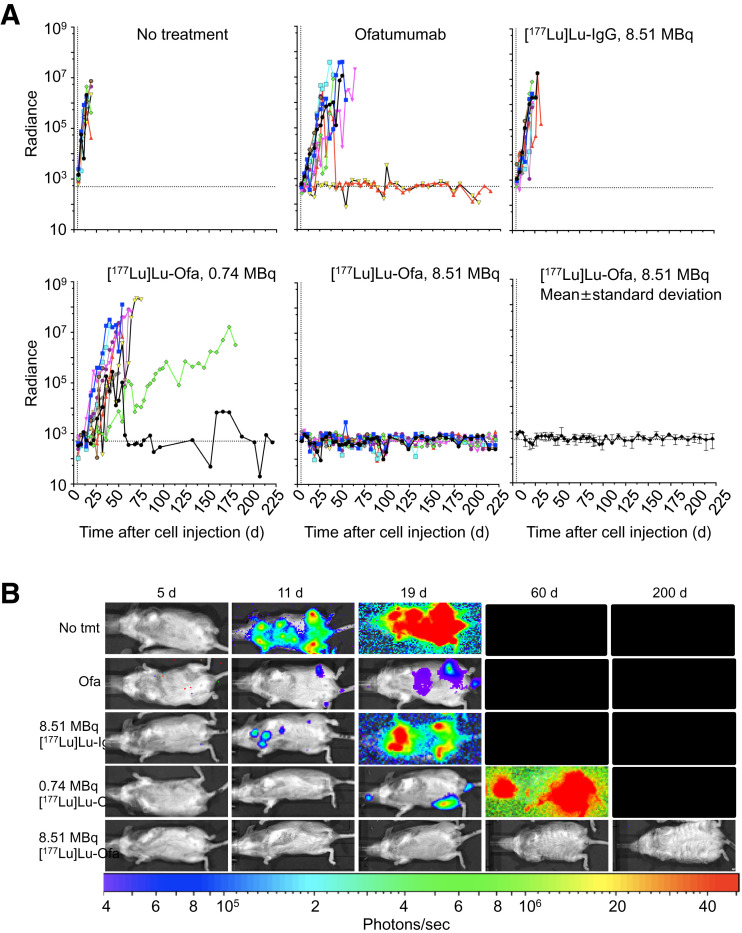
Tumor burden of mice with disseminated Raji-luc cells with therapy initiated 4 d after cell injection. (A) Bioluminescence (10 mice per group). (B) Representative bioluminescence images at indicated days after cell injection. Radiance is photons/s/cm^2^/steradian. Ofa = ofatumumab.

**FIGURE 4.**
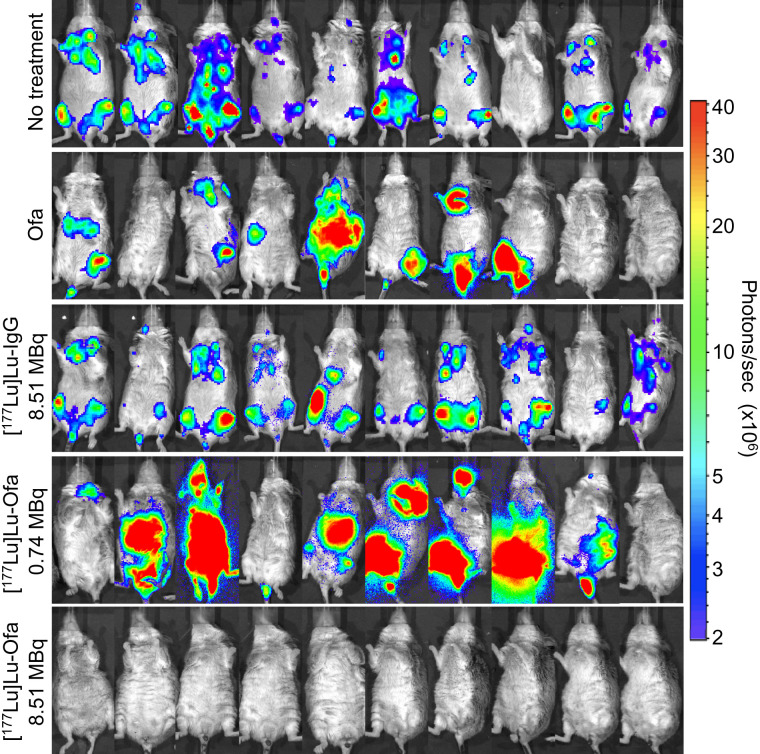
Bioluminescence images of untreated or treated mice with disseminated Raji-luc cells on final imaging event before mice were killed for cause or study termination. Ofa = ofatumumab.

Four days after cell injection, the mice either were left untreated or were treated with native ofatumumab, 8.51 MBq of [^177^Lu]Lu-human IgG1 (345 ± 27 MBq/kg), 0.74 MBq (30 ± 2.2 MBq/kg) of [^177^Lu]Lu-ofatumumab, or 8.51 MBq (345 ± 25.1 MBq/kg) of [^177^Lu]Lu-ofatumumab (10 mice per group). Survival (Fig. 2), weight (Supplemental Fig. 2), and bioluminescence (Fig. 3A) were tracked for 221 d. Representative bioluminescent images at selected time points are shown in Figure 3B, and images of all mice just before they died or were killed for cause or study termination are shown in Figure 4.

The median survival of untreated mice was 19 d, with none surviving beyond 22 d. Unlabeled ofatumumab yielded a median survival of 46 d, superior to untreated mice (Mantel–Cox, *P* < 0.0001), with 1 mouse surviving without weight loss or increased bioluminescence. An 8.51-MBq dose of [^177^Lu]Lu-IgG yielded 0 of 10 surviving mice and a median survival of 25 d, which was not different from that of untreated mice. For all 3 groups, increased bioluminescence and weight loss occurred before death or killing for cause.

A 0.74-MB dose of [^177^Lu]Lu-ofatumumab yielded median survival of 59 d (9/10 mice not surviving), with increased bioluminescence and weight loss before death or killing for cause. This survival was superior to that of untreated mice (Mantel–Cox, *P* < 0.0001) but not to that of mice receiving treatment with unlabeled ofatumumab. Hind-limb paralysis was frequently associated with death or killing for cause (Supplemental Table 3).

Notable therapeutic efficacy resulted from treatment with 8.51 MBq of [^177^Lu]Lu-ofatumumab, with 9 of 10 mice surviving with continuous low bioluminescence (Figs. 3 and 4). This survival was greater than that of untreated mice and of mice treated with unlabeled ofatumumab, 8.51 MBq of [^177^Lu]Lu-IgG, or 0.74 MBq of [^177^Lu]Lu-ofatumumab (Mantel–Cox, *P* < 0.0003 for all comparisons). One mouse succumbed at 117 d, but this death appeared unrelated to tumor burden or therapy as no weight loss or increased bioluminescence occurred. Surviving mice displayed weight loss from 10 to 35 d after cell injection but recovered and gained weight.

**FIGURE 5.**
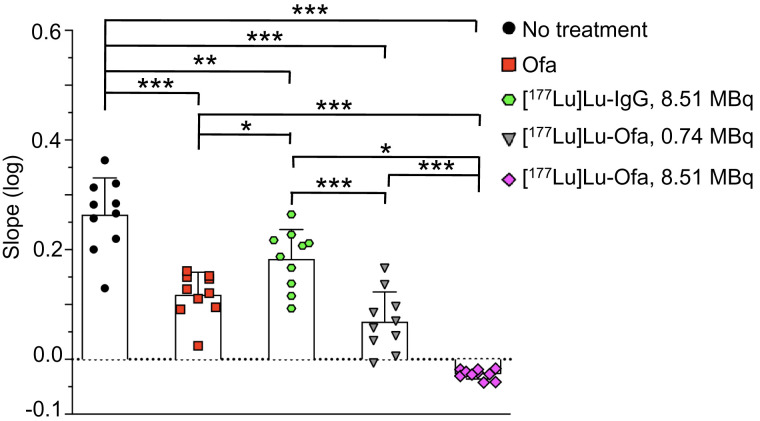
Tumor-cell growth in mice 1–18 d after initiation of therapy. Log of slopes of radiance (photons/s/cm^2^/steradian) over this time are shown as mean ± SD and were analyzed by ANOVA, comparing all samples with each other (10 mice per group). *P < 0.05. **P < 0.005. ***P < 0.0001. Ofa = ofatumumab.

To determine how quickly therapy affected tumor cells, bioluminescence slopes from 1 to 18 d after initiation of therapy were compared (Fig. 5; Supplemental Fig. 3). Compared with no treatment, ofatumumab, 8.51 MBq of [^177^Lu]Lu-IgG, and 0.74 MBq of [^177^Lu]Lu-ofatumumab slowed, but did not eliminate, tumor-cell proliferation. Incontrast, 8.51 MBq of [^177^Lu]Lu-ofatumumab quickly eliminated tumor cells, a finding that was significant compared with no treatment, treatment with unlabeled ofatumumab, treatment with 8.51 MBq of [^177^Lu]Lu-IgG, or treatment with 0.75 MBq of [^177^Lu]Lu-ofatumumab (*P* < 0.05).

## DISCUSSION

Our preclinical studies add to prior work demonstrating the potential of radiolabeled anti-CD20 antibodies to treat NHL. We show that [^177^Lu]Lu-ofatumumab can be produced with high radiochemical yield and purity, excellent affinity, good stability and immunoreactivity, and potent cell killing. Additional advances include using a fully human anti-CD20 and ^177^Lu, which have broad applicability in radiotherapy of cancer. In a model of rapidly progressing disease, we evaluated [^177^Lu]Lu-ofatumumab therapy using dose-response studies and bioluminescence monitoring of tumor-cell burden. A single 8.51-MBq dose of [^177^Lu]Lu-ofatumumab displayed curative efficacy.

Human dosimetry estimates predict that the highest dose from [^177^Lu]Lu-ofatumumab (1.02 mSv/MBq) will be to the heart wall. The relatively radiation-resistant liver and spleen showed 0.36 and 0.48 mSv/MBq, respectively. The predicted dose to red marrow is 0.54 mSv/MBq, and hematologic toxicity likely will be dose limiting in clinical use, as was found with Bexxar, Zevalin, [^177^Lu]Lu-J591 (19), [^177^Lu]Lu-G250 anti-CAIX (20), and [^177^Lu]Lu-rituximab (21). As 2 Sv is a typical maximal dose for acceptable hematologic toxicity without stem cell support, delivering this radiation to the marrow would be tolerable. As there may be patient-to-patient variability with [^177^Lu]Lu-ofatumumab due to cross reactivity with normal CD20-positive cells, our dosimetry data provide guidance for activity administration to humans. Dosimetric estimation could also potentially be obtained using a PET imaging surrogate, such as [^89^Zr]Zr-ofatumumab (9*,*^22^).

The stable in vivo chelation of ^177^Lu by CHX-A″-DTPA-ofatumumab agrees with the results of others using this chelator–radionuclide combination (23*,*^24^). Although it has been suggested that, for stable ^177^Lu chelation, macrocyclic DOTA requires high temperatures incompatible with maintaining antibody function (24*,*^25^), experiments show that this is not the case (26*,*^27^). Thus, CHX-A″-DTPA and DOTA both appear practical for chelation of ^177^Lu to antibodies and antibody fragments.

Others have used [^177^Lu]Lu-anti-CD20 intact antibodies or ^177^Lu-labeled antibody–based radiopharmaceuticals for preclinical and clinical therapy. Ertveld et al. (23), using a single-domain anti-CD20 antibody in immunocompetent mice with CD20-expressing subcutaneous tumors, found a modest therapeutic effect at 140 MBq/mouse; 50 MBq/mouse induced expression of proinflammatory genes, whereas 140 MBq/mouse increased the percentage in the tumor of PD-L1–positive myeloid cells and alternatively activated macrophages. Krasniqi et al. (28) compared a single-domain anti-CD20 antibody with unlabeled rituximab and [^177^Lu]Lu-CHX-A″-DTPA-rituximab in mice with CD20-expressing subcutaneous tumors. All treatments increased survival over no treatment, but [^177^Lu]Lu-CHX-A″-DTPA-rituximab was only slightly better than rituximab. In a phase I/II study of [^177^Lu]Lu-DOTA-rituximab in 31 patients with relapsed or refractory CD20-positive lymphoma, mainly hematologic toxicity was observed, with frequent tumor responses and 8 of 11 patients with follicular lymphoma alive after an 84-mo median follow-up (29).

A major finding of the current study is the high therapeutic efficacy of [^177^Lu]Lu-ofatumumab in a murine model of disseminated lymphoma. Therapy was initiated 4 d after intravenous cell injection, when tumor cells are present individually or as small groups, comparable to micrometastatic or minimal residual disease in humans. An 8.51-MBq dose of [^177^Lu]Lu-ofatumumab reduced tumor burden within about 2 d and eliminated bioluminescence-detectable tumors, with 9 of 10 mice still alive 221 d later. This response was dose-dependent and specific, as 0.74 MBq of [^177^Lu]-Lu ofatumumab and 8.51 MBq of [^177^Lu]-IgG did not extend survival or prevent tumor-cell proliferation. Although attenuation from tissue, skin, and fur means that bioluminescent imaging may not detect a low tumor-cell burden (13), the durability of the response suggests complete elimination of tumor cells by 8.51 MBq of [^177^Lu]Lu-ofatumumab. After initial weight loss, these mice gained weight, suggesting no or low whole-body toxicity. The internalization of ofatumumab after CD20 binding (30) and the residualization of ^177^Lu within the cell may contribute to its therapeutic efficacy. Moreover, the lack of murine sequences in [^177^Lu]Lu-ofatumumab suggests a potential for fractionated therapy or repeated treatments. In an interesting approach, with relatively small subcutaneous tumors of rituximab-resistant Raji cells, Malenge et al. (26) combined [^177^Lu]Lu-lilotomab (anti-CD37) and unlabeled rituximab, with good therapeutic results.

α-particle therapy is another potential approach to treating lymphoma. Using a murine Raji-cell disseminated lymphoma model, [^213^Bi]Bi-rituximab (t_½_, 45.6 min) was typically curative when tumor burden was low (4 d after cell injection) but not when it was higher (18), perhaps because of lack of time to target larger tumor masses before decay. Similarly, [^149^Tb]Tb-rituximab (t_½_, 4.2 h) therapy initiated 2 d after Daudi-cell intravenous injection increased survival (31). A 1F5 anti-CD20 antibody with chelated ^211^At (t_½_, 7.2 h) was 80% curative when injected 6 d after intravenous cell injection with supporting stem-cell transplantation but only slowly reduced the growth of subcutaneous tumors (32). On the basis of these results and on the multiday tumor-targeting pharmacokinetics of intact antibodies, radioimmunotherapy of larger tumor masses with intact antibodies will likely be most successful using radioisotopes that permit tumor localization before decay, including ^177^Lu or α-particle–emitting ^225^Ac with its 10-d half-life.

Our studies add to the literature demonstrating the effectiveness of ^177^Lu-radiopharmaceuticals in cancer therapy. We found remarkable effectiveness in micrometastatic disease, and the 6.6-d half-life and multiple–cell-diameter killing range of ^177^Lu suggests that [^177^Lu]Lu-ofatumumab may be effective against larger tumors.

Although initial anti-CD20 radioimmunotherapies showed limited commercial success for several reasons, we suggest that a reevaluation of next-generation β- and α-particle therapies is in order. [^177^Lu]Lu-ofatumumab CD20-targeted radioimmunotherapy may be an effective approach for therapy of NHL or other CD20-expressing diseases.

## CONCLUSION

Chx-A″-DTPA-ofatumumab stably chelates ^177^Lu in vitro and in vivo, and [^177^Lu]Lu-Chx-A″-ofatumumab effectively targets CD20-expressing tumor xenografts. In a mouse model of disseminated human lymphoma, therapy with [^177^Lu]Lu-ofatumumab showed curative therapeutic efficacy.

## DISCLOSURE

This study was supported in part by the National Institutes of Health (R01CA240711, R01CA229893, and R01CA201035 to Daniel Thorek), the Children’s Discovery Institute (MC-II-2021-961 to Diane Abou), and the NIGMS (9995P41GM103422 to the Washington University Biomedical Mass Spectrometry Resource). Richard Wahl is on the scientific advisory board of Clarity Pharmaceuticals, Voximetry, and Seno Medical; has stock options in Clarity Pharmaceutical and Voximetry; receives honoraria from Bristol Myers Squibb, Actinium Pharmaceuticals, Jubilant Draximage, and ITM; and receives research support from Actinium Pharmaceuticals, BMS, Bayer, Siemens, and White Rabbit AI. Diane Abou and Daniel Thorek have an advisory board role for, and own stock in, Diaprost AB and Pharma15. Richard Laforest is a consultant to Curium Pharmaceuticals. No other potential conflict of interest relevant to this article was reported.

## Notes

KEY POINTS **QUESTION:** Can [^177^Lu]Lu-anti-CD20 ofatumumab be produced and effectively treat disseminated human NHL in a murine model? **PERTINENT FINDINGS:** [^177^Lu]Lu-ofatumumab specifically killed lymphoma cells in vitro, was cytotoxic in vivo as assessed by bioluminescence imaging, and was curative of disseminated human lymphoma in the murine model. Dosimetry estimates support the feasibility of human translation. **IMPLICATIONS FOR PATIENT CARE:** [^177^Lu]Lu-ofatumumab has exceptional potential for CD20-targeted radioimmunotherapy of patients with NHL.
